# Association between circulating cell-free mitochondrial DNA and inflammation factors in noninfectious diseases: A systematic review

**DOI:** 10.1371/journal.pone.0289338

**Published:** 2024-01-19

**Authors:** Min Zhou, Hao Zhang, Xin Xu, Hairen Chen, Baiwen Qi

**Affiliations:** 1 Department of Orthopeadics Trauma and Microsurgery, Zhongnan Hospital of Wuhan University, Wuhan, Hubei, China; 2 Department of Cardiology, Zhongnan Hospital of Wuhan University, Wuhan, Hubei, China; Phramongkutklao College of Medicine, THAILAND

## Abstract

**Objective:**

This study aimed to assess the correlation between the circulating cell-free mitochondria DNA and inflammation factors in noninfectious disease by meta-analysis of data from eligible studies.

**Materials and methods:**

Through a comprehensive searching of pubmed, embase, web of science, cochrane from establishment of the database to October 31, 2022, studies were selected that investigated the association of circulating cell free mitochondria DNA with inflammatory factors in non-infectious diseases. Studies that met the inclusion criteria and were published in English or Chinese were included. Data of each correlation coefficients were extracted from the paper and 95% confidence intervals were calculated. Sensitivity and heterogeneity tests were carried out for each data.

**Results:**

A total of 660 articles were retrieved and 22 were included in this meta-analysis, including 2600 patients. A fixed effects model was employed to examine ISS and IL-8, others were analyzed using random effects models. The correlation coefficient between mtDNA and ISS score was 0.37 (95%CI = [0.232;0.494]), p<0.0001, heterogeneity I^2^ = 46%, p = 0.11). The correlation coefficients between mtDNA and inflammatory factors are as follows: TNFα, 0.405 [(95%CI = [0.253;0.538], p<0.0001, heterogeneity I^2^ = 77%, p = 0.0001]. IL-6, 0.469 [(95%CI = [0.296;0.612]), p = 0.0001, heterogeneity I2 = 93%, p<0.0001]. CRP, 0.333[(95%CI = [0.149;0.494]), p = 0.005, heterogeneity I2 = 85%, p<0.0001]. IL-8, 0.343[(95%CI = [0.233;0.524]), p = 0.001, heterogeneity I^2^ = 50%, p = 0.09]. PCT, 0.333 [(95%CI = [0.06;0.64]), p = 0.09,heterogeneity I^2^ = 64%,p = 0.06]. There were no significant publication bias for TNFα, IL-6 and CRP.

**Conslusion:**

Circulating cell free mtDNA was moderate positively correlated with the expression of inflammatory factors and the degree of trauma.

## Introduction

Non-infectious diseases such as acute trauma and surgery often lead to tissue damage and inflammatory responses. These diseases can activate neutrophils, impaired endothelial cell function through releasing proteolytic enzyme, oxygen free radical [1]. These reaction increases the permeability of capillaries and extensive infiltration of inflammatory cells, then develop into systemic inflammatory response syndrome, and further to impair organ function, eventually result in multiple organ dysfunction [2]. How to assess severtiy of noninfectious inflammatory response caused by non-infectious diseases is an urgent clinical problem to be solved.

According to recent study, acute trauma and surgery results in cell membranes rapture, following the contents of cell spill out onto extracellular milieu. Massive release of DAMP (damage associated molecular patterns) damages adjacent cells, causing more cell necrosis and releasing more DAMP [3]. Mitochondrial DAMP released into circulate was identified by cellular surface receptor and triggered a serious inflammation responds [4]. Mitochondria DNA is a common DAMP and has ability to active multiple immune cells, such as monocytes, macrophages, and then induce release of IL-6, TNFα, IL-1, which result in system inflammation [5, 6]. After mtDNA is recognized by TLR9, interaction of TLR9 and Myd88 activate MAPK and NFƙB, leading to produce proinflammation factors such as IL-1, IL-6 and TNFα [7]. MtDNA activates NLRP3 recruited by mitochondria,then induce the production of caspase-1,which produce active IL-1β through cleaving inactive pro-IL-1β and promote IL-18 expression [8]. MtDNA induce IFN expression by activating cGAS-STING-TBK1 pathway [9]. Plasma mitochondrial DNA (mtDNA) level in trauma patients was prominent higher than non-trauma patients. The level of mtDNA in patients who developed Systemic inflammatory response syndrome (SIRS) was significantly higher than that in non-SIRS patients [10]. Although experimental and clinical studies have shown that mtDNA concentration is associated with inflammatory responses, the relationship between mtDNA concentration and inflammatory factors remains unclear.

Several studies have shown that the content of mtDNA in plasma is positive correlated with the expression of IL-6, TNFα. Other studies have shown that DNA is negatively or no correlated with inflammatory factors. So, the result remains controversial [11, 12]. In addition, the inflammatory response caused by infection also increase the expression of IL-1, IL-6 and other inflammatory factors through interaction of PAMP and TLR [13].

Therefore, in order to avoid the interference of infection, we performed this meta-analysis in order to uncover the correlation between plasma mtDNA and inflammation factors in noninfectious diseases, such as acute trauma, surgery, AMI, dialysis, diabetes.

## Materials and methods

The present meta-analysis was reported according to the Preferred Reporting Items for Systematic reviews and Meta-Analyses (PRISMA) Statement [14].

### Inclusion criteria

If article met the following criteria, it was selected for inclusion: ① the study subject were patients; ② the disease studied was non-infectious; ③ the outcome was the relationship between circulating mtDNA level and inflammatory factors, such as TNF-α, IL-6, IL-1β, C-reactive protein (CRP), IL-8, procalcitonin (PCT), NFƙB, and Injury Severity Score (ISS); ④ the article was published in a peer-reviewed scientific journal as a full paper; ⑤ prospective, retrospectively and cross-section studies

### Exclusion criteria

The articles were excluded according to following criteria: ① The research subjects were animals or cell; ② The same set of data was published repeatedly; ③ The articles not provide the data of correlation coefficient and number of cases and data was not available after contacting the author; ③ The full articles were unavailable. ④ reviews, case reports, abstracts of meetings and comments.

### Literature research

The following databases were searched by two independent observer from establishment of the database to October 31, 2022: Pubmed, Embase, Cochrane library, Web of Science, Cochrane Library and China National Knowledge Infrastructure (CNKI). The searching terms included “mtDNA” OR “mitochondria DNA” OR “mitochrondria deoxyribonucleic acid” And “TNFα” OR “IL-6” OR “IL-1β” OR “ISS” OR “CRP” OR “PCT” OR “NFƙB”. The language of published articles is limited to Chinese and English. Articles searched in databases were published prior to and including October 2022. All retrieved articles were manually crosscheck for titles and abstracts, and full-text inspection followed according to selection criteria.

### Evaluation of literature quality

The included studies were independently scaned by two observers (MZ and Hr Ch) using the The Agency for Healthcare Research and Quality. The useful data extracted from each study based on a table, included the following: authors, the nation of origin, the year of publication, the number of the patients, Pearson or Spearman correlation coefficient (r). Disagreements between the two reviewers were resolved by a majority opinion after a third reviewer (Bw Qi) assessed all involved items.

### Extraction of data

After selected by reading abstract, included articles were independently evaluated by two investigators, and data were extracted base on a standardized form. The variables included: first author, publication year, study design, study period, geographic region, r value and total sample size. The correlation coefficient between mtDNA and inflammation factors, such as TNF-α, IL-6, CRP, IL-8, PCT, IL-10, IL-1, lactate and ISS score were recorded detail. If the data provided by the article is incomplete, contact the author to obtain complete data.

Pearson correlation coefficients published in article were converted into Spearman correlation coefficients [15]. Fisher’s transformation, changing Spearman correlation coefficients to a Z-score, was used to compare [16].

### Statistical analysis

After suitable transformation, the corresponding data from each study was combined with a random effect model [17].

Statistical heterogeneity was assessed by the chi-square test on Q statistic, which was quantified by the I-square values, assuming that I-square values 50 were nominally assigned as moderate estimates [18]. The fixed-effect model was used after no significant heterogeneity was monitored. To investigate potential sources of heterogeneity, stratified analyses was performed to find any possible sources. Sensitivity analysis was also performed by excluding each study at a time to assess whether one or more studies influenced the overall results. Publication bias was assessed first by visually inspecting the distribution of observed studies on a funnel plot [19]. And P ≤0.05 was indicated the presence of statistically significant.

## Results

### Results of literature retrieval and screening

**Fig 1:**
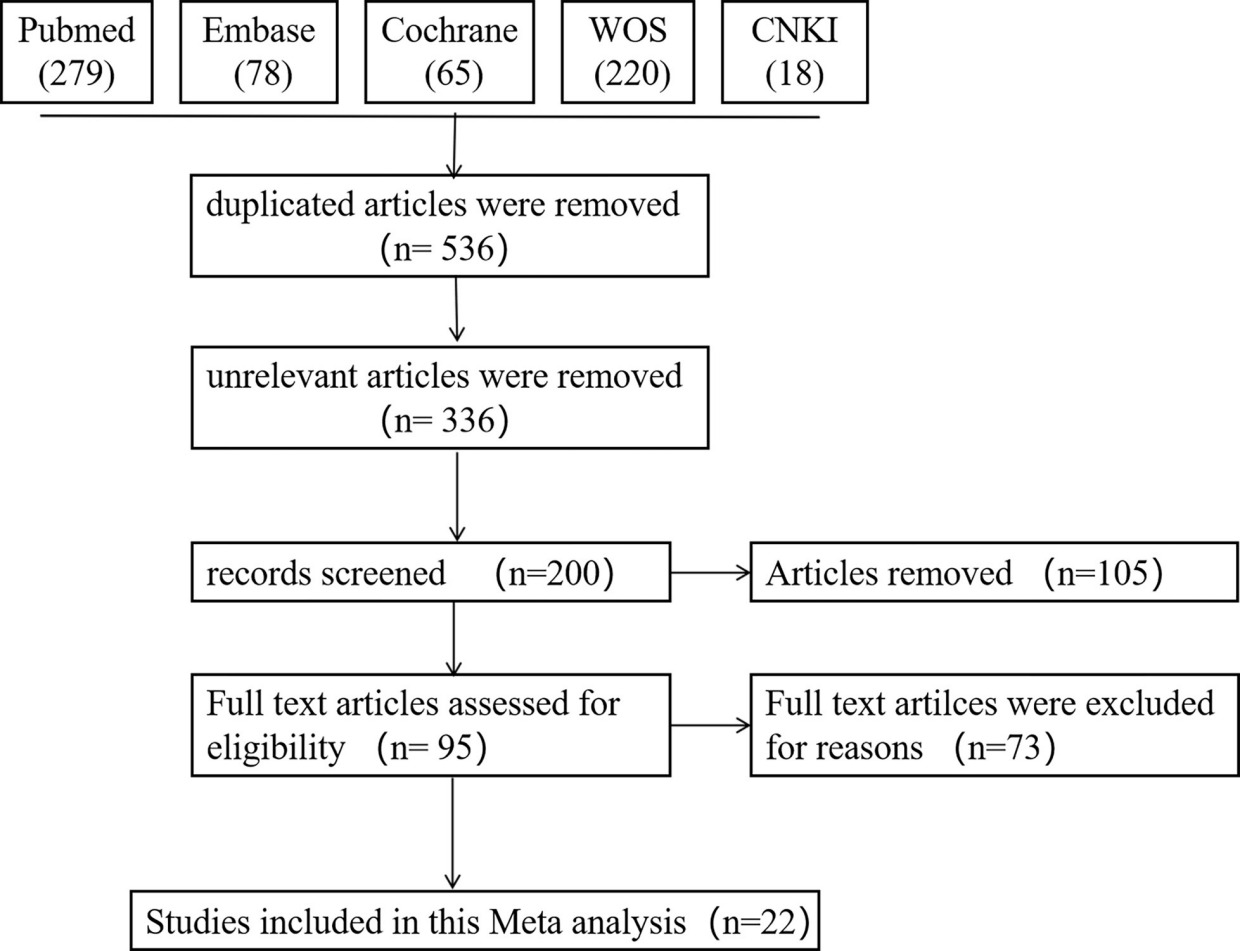
Flowchart of study selection process.

A total of 660 articles were retrieved by searching databases. 460 irrelevant articles were eliminated by reading abstracts due to duplications (n = 124), non-revelance(n = 336). 105 articles of review were excluded. 5 articles were excluded for which full text cannot be obtained. 10 articles that trauma combined with infection, 54 articles with inconsistent outcome, 4 animal experiments were excluded by reading the full text. In the end, a total of 22 papers were included (19 in English, 3 in Chinese; Fig 1). The basic information of the included literature was summarized in Table 1. All literatures were scored using The Agency for Healthcare Research and Quality (Table 2). The quality of the literature is acceptable.

**Table 1:** Charectersitcs of inclued studies.

Study	Year	N	Diagnosis	Age	Design
Emmanuel [20]	2020	80	Postsurgical	Adult	prospectively
Fan [21]	2020	962	Cardiac surgery	Adult	retrospective
Gu [10]	2013	86	Trauma	Adult	prospectively
Guo Enwei [22]	2020	47	Trauma	Adult	prospectively
Jia [23]	2016	136	AMI	Adult	prospectively
Lam [24]	2004	38	Trauma	Adult	prospectively
Qin [25]	2015	38	Cardiac surgery	Adult	prospectively
Qin [26]	2017	67	AMI	Adult	prospectively
Qin [27]	2016	46	Cardiac surgery	Adult	prospectively
Yamanouchi [28]	2013	60	Trauma	Adult	prospectively
Wang [29]	2019	110	Trauma	Adult	prospectively
Xie [30]	2019	189	peritoneal dialysis	Adult	prospectively
Zhang [31]	2017	156	trauma	Adult	prospectively
Bulgakova [32]	2021	65	Lung cancer	Adult	prospectively
Cañizales [33]	2018	68	Diabetes	Adult	prospectively
Fan [34]	2022	97	Sarcopenia	Adult	Cross-sectional study
Gonçalves [35]	2021	32	Late-life depression	Adult	prospectively
Kageyama [36]	2018	97	Major depressive disorder	Adult	prospectively
Lee [37]	2014	94	Fat Accumulation	Adult	prospectively
Peng [38]	2019	33	Autoimmune encephalitis	Adult	prospectively
Xu [39]	2017	48	Cardiopulmonary bypass	Infant	prospectively
Zhong [40]	2022	52	Hemodialysis	Adult	cross-sectional study

AMI: acute myocardium infarction

**Table 2:** Methodological quality of included studies according to the Agency for Healthcare Research and Quality (AHRQ).

Study	Year	Score
**Emmanuel**	2020	7
**Fan**	2020	3
**Gu**	2013	6
**Guo**	2020	7
**Jia**	2016	7
**Lam**	2004	7
**Qin**	2015	6
**Qin**	2016	6
**Qin**	2017	5
**Satoshi**	2013	7
**Wang**	2019	6
**Xie**	2016	6
**Zhang**	2017	7
**Bulgakova**	2021	6
**Cañizales**	2018	6
**Fan**	2022	5
**Gonçalves**	2021	6
**Kageyama**	2018	7
**Lee**	2014	5
**Peng**	2019	7
**Xu**	2017	5
**Zhong**	2022	6

### Correlation between mtDNA and inflammation factors

**Fig 2:**
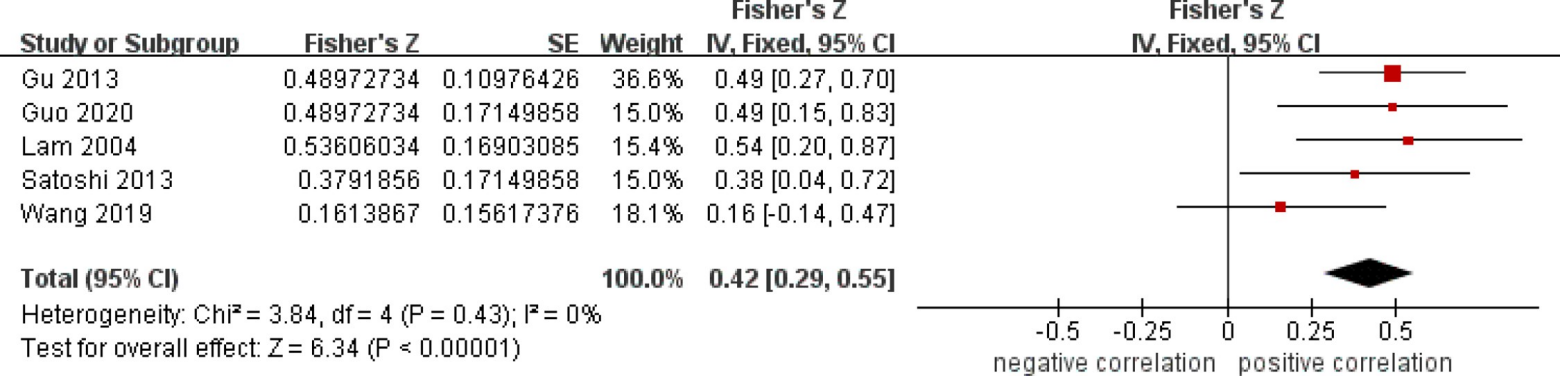
Forest plots of correlation coefficients between mtDNA and ISS score.

**Fig 3:**
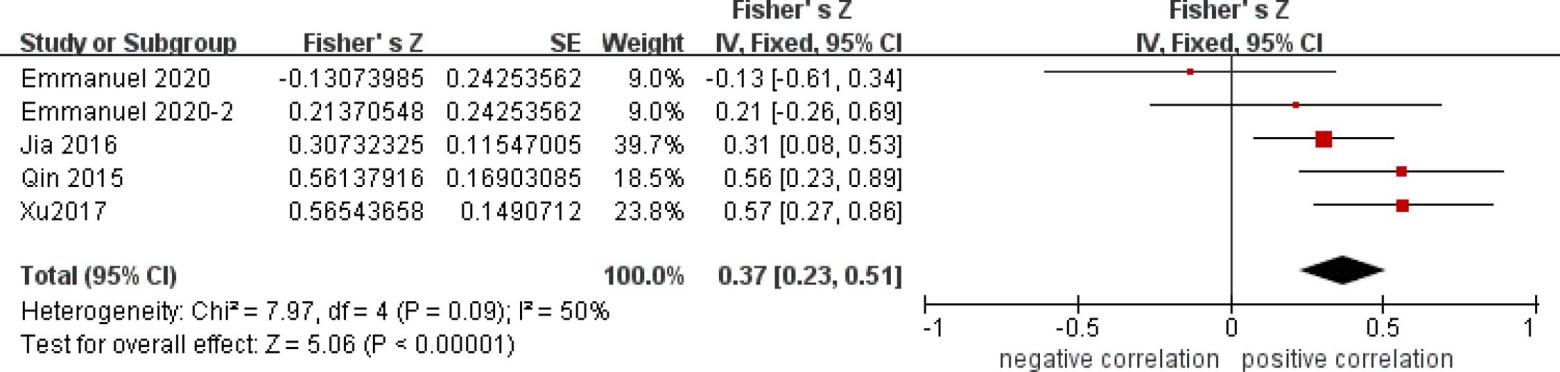
Forest plots of correlation coefficients between mtDNA and IL-8.

**Fig 4:**
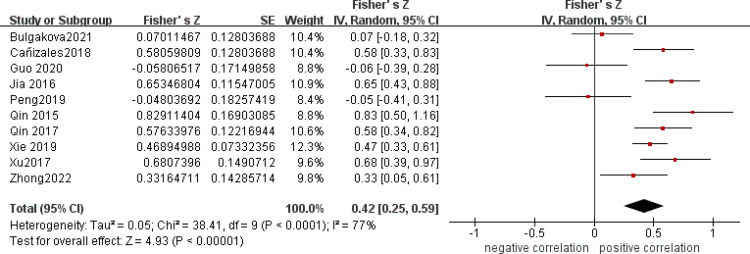
Forest plots of correlation coefficients between mtDNA and TNF-α.

**Fig 5:**
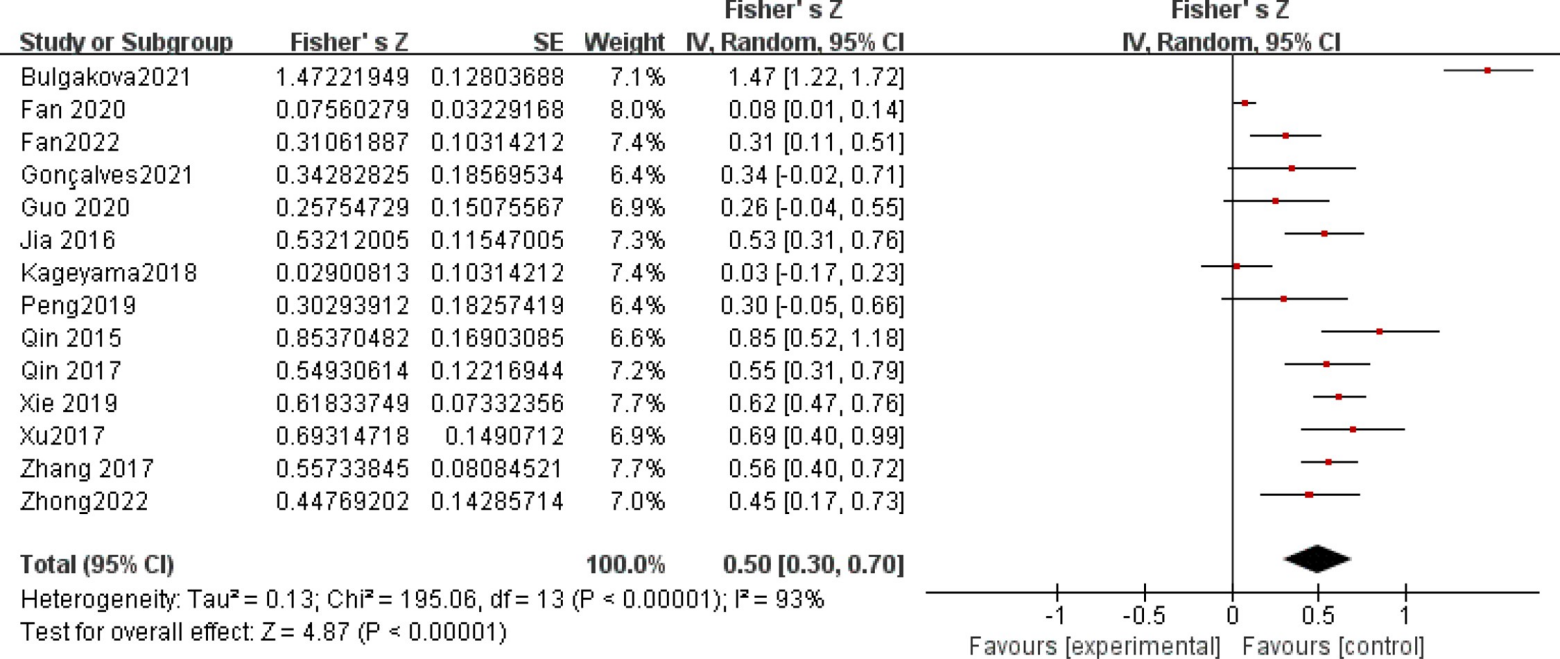
Forest plots of correlation coefficients between mtDNA and IL-6.

**Fig 6:**
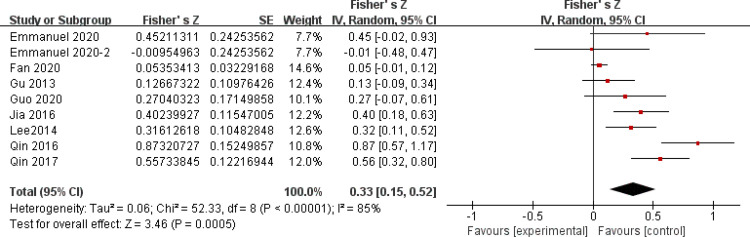
Forest plots of correlation coefficients between mtDNA and CRP.

**Fig 7:**
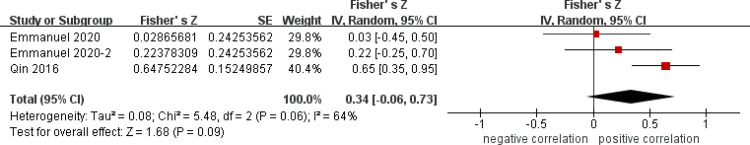
Forest plots of correlation coefficients between mtDNA and PCT.

**Fig 8:**
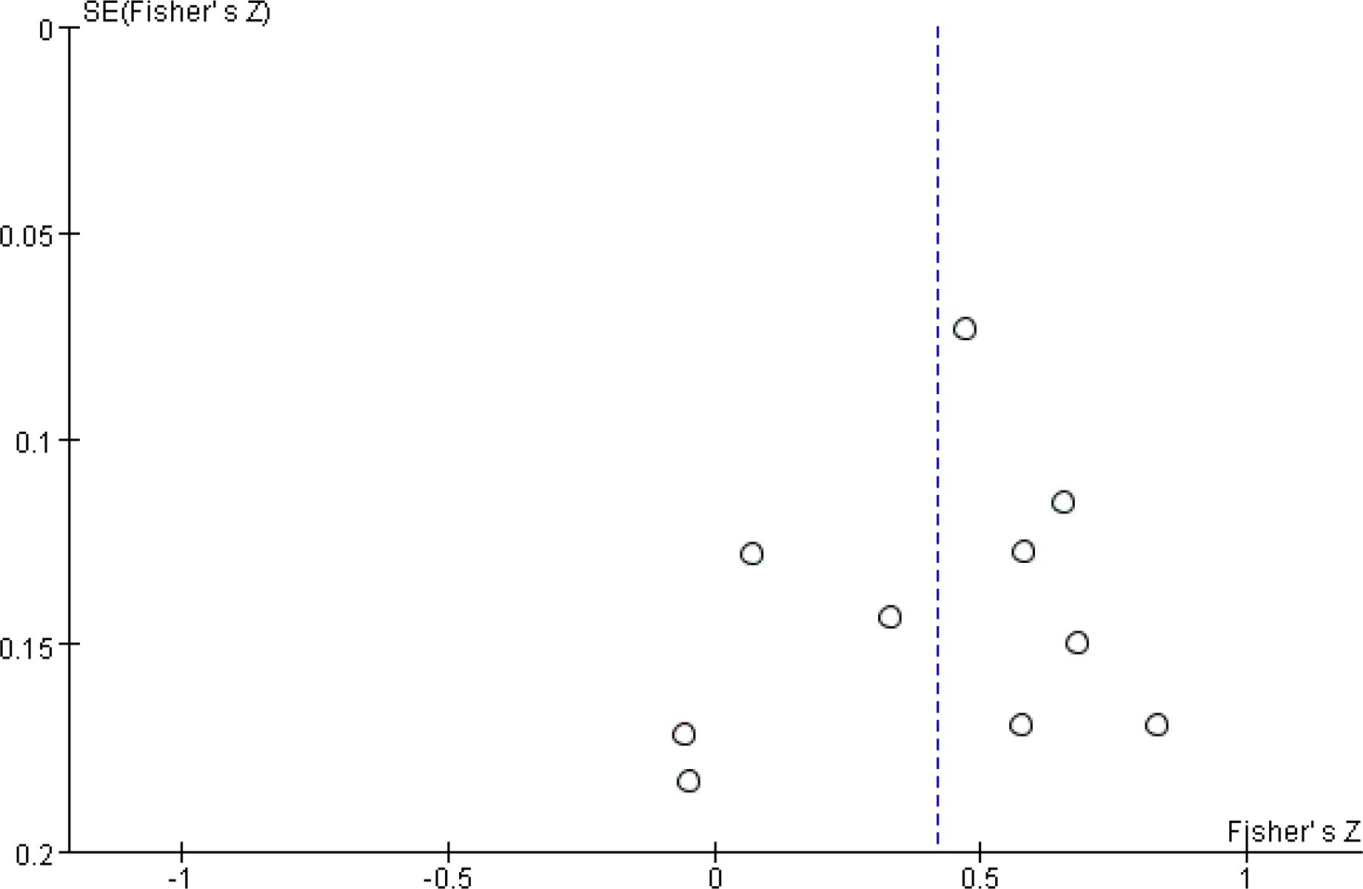
Funnel plot for TNF-α.

**Fig 9:**
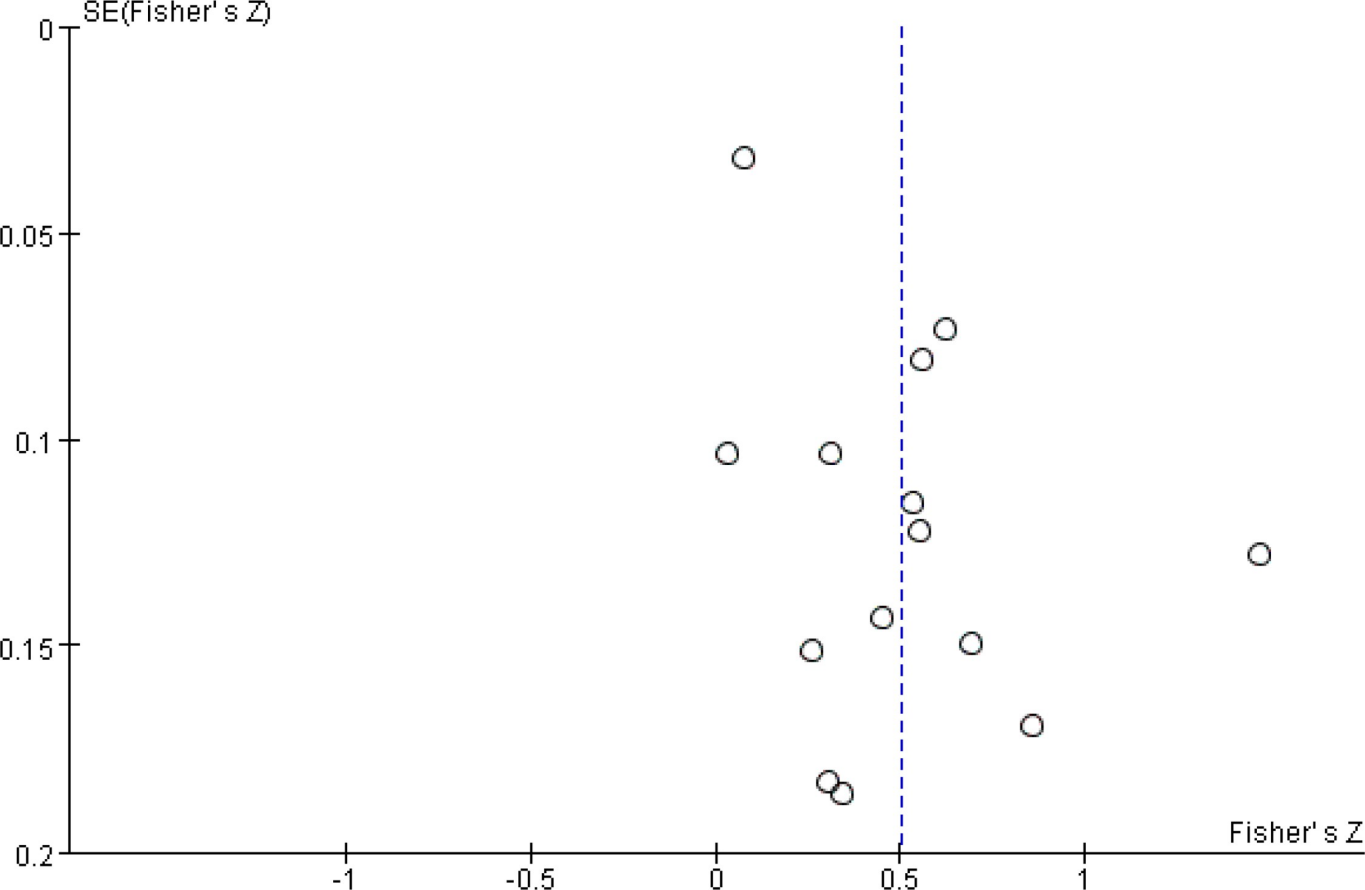
Funnel plot for IL-6.

After a heterogeneity test, there were no notable heterogeneity among ISS score and IL-8, so fixed effects model was used for meta-analysis. The results showed that plasma mtDNA was moderately positive correlation with ISS score (r = 0.37(0.232, 0.494))(Fig 2) and IL-8 (r = 0.343(0.133, 0.524)) (Fig 3). After heterogeneity test, we found there was great heterogeneity among TNFα (Fig 4), IL-6 (Fig 5), CRP (Fig 6) and PCT (Fig 7), so the random effects model was used. The results of funnel plot showed no evidence of obvious publication bias of TNFα (Fig 8) and IL-6 (Fig 9), and p value of egger’s test for TNFα, IL-6 and CRP was 0.225, 0.349 and 0.631, respectively. Plasma mtDNA was moderately positive correlation with TNFα (Fig 2), IL-6(Fig 9) and CRP(Fig 6). There was no correlation between plasma mtDNA and PCT (p = 0.09) (Fig 7). (The results of meta analysis were summarized in Table 3)

**Table 3:** Summery of meta analysis for factors.

Items	No. of experiments	Fisher Z	r(95%CI)	I^2^	P value (heterogeneity)	Z	P value
ISS(Fig 2)	5	0.42(0.29, 0.55)	0.37(0.232, 0.494)	46%	0.11	4.99	0.0001
TNFɑ(Fig 4)	10	0.42(0.25, 0.59)	0.405(0.253, 0.538)	77%	0.0001	4.93	0.0001
IL-6(Fig 5)	14	0.50(0.30, 0.70)	0.469(0.296, 0.612)	93%	0.0001	4.81	0.0001
CRP(Fig 6)	9	0.33(0.15, 0.52)	0.333(0.149, 0.494)	85%	0.0001	3.46	0.0005
IL-8(Fig 3)	5	0.37(0.23, 0.51)	0.343(0.233, 0.524)	50%	0.09	3.19	0.001
PCT(Fig 7)	3	0.34(-0.06, 0.73)	0.333(0.06, 0.64)	64%	0.06	1.68	0.09

The GRADE quality of evidence for TNFα, IL-6 and CRP were judged to be high, ISS and IL-8 were judged to be moderate. GRADE evidence quality was summarized in Table 4.

**Table 4:** GRADE evaluation for inflammation factors.

Certainty assessment							№ of patients		Effect		Certainty	Importance
№ of studies	Study design	Risk of bias	Inconsistency	Indirectness	Imprecision	Other considerations	[干预]	[对照]	Relative (95% CI)	Absolute (95% CI)		
**ISS**												
5	observational studies	not serious	not serious	not serious	not serious	publication bias strongly suspected strong association dose response gradient^a^	242/242 (100.0%)	149/149 (100.0%)	**Fisher’s Z 0.39** (0.24 to 0.54)	**— per 1,000** (from—to—)	⨁⨁⨁◯ Moderate	IMPORTANT
**TNF**												
10	observational studies	not serious	not serious	not serious	not serious	strong association dose response gradient	642 cases 255 controls		**Fisher’ s Z 0.42** (0.25 to 0.59)	-	⨁⨁⨁⨁ High	CRITICAL
							-	0.0%		**— per 1,000** (from—to—)		
**IL-6**												
14	observational studies	not serious	not serious	not serious	not serious	strong association dose response gradient	1963 cases 290 controls		**Fisher’ s Z 0.5** (0.3 to 0.7)	-	⨁⨁⨁⨁ High	CRITICAL
							-	0.0%		**— per 1,000** (from—to—)		
**CRP**												
9	observational studies	not serious	not serious	not serious	not serious	strong association dose response gradient	1413 cases 181 controls		**Fisher’ s Z 0.33** (0.15 to 0.52)	-	⨁⨁⨁⨁ High	CRITICAL
							-	0.0%		**— per 1,000** (from—to—)		
**IL-8**												
5	observational studies	serious^a^	not serious	not serious	not serious	all plausible residual confounding would suggest spurious effect, while no effect was observed dose response gradient	204 cases 98 controls		**Fisher’ s Z 0.37** (0.23 to 0.51)	-	⨁⨁⨁◯ Moderate	CRITICAL
							-	0.0%		**— per 1,000** (from—to—)		
**PCT**												
3	observational studies	serious^b^	not serious	not serious	not serious	publication bias strongly suspected all plausible residual confounding would suggest spurious effect, while no effect was observed^a^	86 cases 40 controls		**Fisher’ s Z 0.34** (-0.06 to 0.73)	-	⨁◯◯◯ Very low	
							-	0.0%		**— per 1,000** (from—to—)		

## Discussions

### Correlation analysis results on mtDNA and inflammation

The purpose of this Meta analysis study was to reveal the correlation between circulating mtDNA content and inflammatory response induced by non-infectious diseases. We excluded inflammatory responses caused by infection, because infection resulted in severe inflammatory responses through other pathways, which would affect the accuracy of the results. The results of this study showed that mtDNA content was correlated with ISS score, TNF-α, IL-6, CRP and IL-8 content, but not with PCT content. This study provides evidence for mtDNA as a molecular marker for predicting inflammation.

When cells are damaged and mitochondria are destroyed, mtDNA is released into the blood. After trauma, compared with non-MODS patients, patients with MODS had significantly higher blood mtDNA concentrations [41]. The content of mtDNA in trauma patients was higher than that in normal control group at before operation, during operation and 3 days after operation. At the same time, the concentration of mtDNA in patients who developed SIRS was significantly higher than that in patients without SIRS. The concentration of mtDNA was correlated with SIRS [10, 42]. Animal experimental studies on hip fractures in rats showed that the concentration of mtDNA increased significantly after surgery, and its concentration was significantly correlated with inflammatory factors IL-10 and TNF-α [43]. The above studies showed that after trauma or surgery, a large amount of mtDNA is released into the blood. And plasma mtDNA levels correlate with injury and disease severity. The results of this study also showed that the circulating mtDNA levels was positively moderate correlated with the score of ISS. This results indicate that circulating mtDNA was positively correlated with the severity of trauma.

Studies have shown that mtDNA promotes TNFα expression. During intestinal ischemia-reperfusion injury, MtDNA-STING signaling pathway promotes intestinal endothelial cell necrosis; in addition, mtDNA promotes the expression of IFN and TNF-α to induce intestinal necrosis [44]. Timmermans et al. [45] study have shown that there were no relationship between mtDNA and TNFα, IL-6, IL-8 in sepsis shock patients. Plasma mtDNA was positive correlation with TNFα in diabetes patients by multiple linear regression [46]. The present meta analysis have confirmed that plasma mtDNA is moderate positive correlation with TNFα.

Zhang et al. showed that in trauma patients with sepsis, mtDNA was negatively correlated with organ function, and was negatively correlated with both IL-6 and IL-10 [47]. In a study of kidney damage caused by SIRS, the results showed that the level of mtDNA in SIRS patients was significantly higher than that in non-SIRS patients. But there was no correlation between mtDNA and IL-6 and IL-8 [9]. Bao et al study showed that plasma mtDNA was strong positive correlation with IL-6 and strong negative correlation with IL-10 in rat hip fracture [48]. Puskarich et al. [11] study showed that plasma mtDNA was negative correlation with IL-6 and IL-10 in sepsis and sepsis shock patients. In diabetes, plasma mtDNA was moderate positive correlation with IL-6 [46]. The above studies indicate that the correlation between mtDNA and IL-6 is uncertain. This study have confirmed that plasma mtDNA is positive correlation with IL-6 in noninfectious disease.

Studies have shown that mtDNA is associated with CRP expression in infectious disease. Results of Huang et al. study showed that plasma mtDNA was significantly positively correlated with CRP in acute respiratory distress syndrome [49]. However, studies have showed that plasma mtDNA was no correlation with CRP in sepsis patients [50]. This meta-analysis showed that in non-infectious diseases, the expression of plasma mtDNA is correlated with the expression of CRP.

### Heterogeneity

The results suggest that TNFα, IL-6 and CRP have distinct heterogeneity, so we analyze the reasons for the heterogeneity. First, the diseases included in the studies were diverse and lacked similarity. Because this study is mainly aimed at non-infectious diseases, the types of diseases include fractures, trauma, surgery, myocardial infarction, hemodialysis and other diseases. The bigger the difference in diseases, the larger the difference in heterogeneity. Second, the quality of the original research was inconsistent. The AHRQ score of the included literature were mostly 5 to 7 points, and the overall quality was not high, resulting in high heterogeneity. Third, different data processing methods also bring some heterogeneity

### Limitations

This meta analysis comes with several limitations. Firstly, the sample size of each included study was small. Except for Fan2020, where the number of cases is 962, the number of cases in the other studies ranges from 17 to 107, which may weaken the reliability of the conclusions. Secondly, In the original research, some articles only give conclusions, and do not provide original data or correlation coefficients, so they cannot be included, which may lead to publication bias. Thirdly, this meta analysis has not been registered online, which may lead to potential bias.

In conclusion,although there are some limitations of this study, the current evidence shows that mtDNA is related to the degree of trauma and the expression of inflammatory factors CRP, IL-6, TNF-α, and IL-8 and play an important role in the inflammatory response. Future research is need to identify clinical value of mtDNA for indicating inflammatory response.

## Supporting information

S1 Checklist PRISMA 2020 checklist. (DOCX)

S1 Data (XLSX)

## References

[1] Relja B, Land WG (2020) Damage-associated molecular patterns in trauma. Eur J Trauma Emerg Surg 46:751–775. doi: 10.1007/s00068-019-01235-w 31612270 PMC7427761

[2] Vourc’h M, Roquilly A, Asehnoune K (2018) Trauma-induced damageassociated molecular patterns-mediated remote organ injury and immunosuppression in the acutely ill patient [J]. Front Immunol 9: 1330. 29963048 10.3389/fimmu.2018.01330 PMC6013556

[3] Thurairajah K, Briggs GD, Balogh ZJ (2018) The source of cell-free mitochondrial DNA in trauma and potential therapeutic strategies. Eur J Trauma Emerg Surg 44(3):325–334. doi: 10.1007/s00068-018-0954-3 29633007 PMC6002458

[4] Lopez-Armada MJ, Riveiro-Naveira RR, Vaamonde-Garcia C. and Valcarcel-Ares MN (2013) Mitochondrial dysfunction and the inflammatory response. Mitochondrion 13, 106–118. doi: 10.1016/j.mito.2013.01.003 23333405

[5] Zhang Q, Raoof M, Chen Y (2010) Circulating mitochondrial DAMPs cause inflammatory responses to injury [J]. Nature 464: 104–107. 20203610 10.1038/nature08780 PMC2843437

[6] West AP, Shadel GS (2017) Mitochondrial DNA in innate immune responses and inflammatory pathology [J]. Nat Rev Immunol 17: 363–375. 28393922 10.1038/nri.2017.21 PMC7289178

[7] Peiseler M, Kubes P (2018) Macrophages play an essential role in trauma-induced sterile inflammation and tissue repair. Eur J Trauma Emerg Surg 44:335–349. doi: 10.1007/s00068-018-0956-1 29666944

[8] Guo H, Callaway JB, Ting JPY (2015) Inflammasomes: mechanism of action, role in disease, and therapeutics [J]. Nat Med, 21(7): 677–687. 26121197 10.1038/nm.3893 PMC4519035

[9] Gao D, Wu J, Wu YT, (2013). Cyclic GMP-AMP synthase is an innate immune sensor of HIV and other retroviruses [J]. Science, 341(6148): 903–906. 23929945 10.1126/science.1240933 PMC3860819

[10] Gu X, Yao Y, Wu G, Lv T, Luo L, Song Y (2013) The plasma mitochondrial DNA is an independent predictor for post-traumatic systemic inflammatory response syndrome. PLoS One 8:e72834. doi: 10.1371/journal.pone.0072834 23977360 PMC3748121

[11] Puskarich MA, Shapiro NI, Trzeciak S, Kline JA, Jones AE (2012) Plasma levels of mitochondrial DNA in patients presenting to the emergency department with sepsis. Shock. 38(4):337–40. doi: 10.1097/SHK.0b013e318266a169 22777124 PMC3664931

[12] Jansen MPB, Pulskens WP, Butter LM, Florquin S, Juffermans NP, Roelofs JJTH, et al. (2018) Mitochondrial DNA is Released in Urine of SIRS Patients With Acute Kidney Injury and Correlates With Severity of Renal Dysfunction. Shock. 49:301–310. 28837526 10.1097/SHK.0000000000000967

[13] Faix JD (2013) Biomarkers of sepsis. Crit Rev Clin Lab Sci;50(1):23–36. doi: 10.3109/10408363.2013.764490 23480440 PMC3613962

[14] Moher D, Liberati A, Tetzlaff J, Altman DG. PRISMA Group (2009) Preferred reporting items for systematic reviews and meta-analyses: the PRISMA statement. J Clin Epidemiol 62:1006–12. doi: 10.1016/j.jclinepi.2009.06.005 19631508

[15] Chalkidou A, Landau DB, Odell EW, Cornelius VR, ODoherty MJ (2012) Correlation between Ki-67 immunohistochemistry and 18F-Fluorothymidine uptake in patients with cancer: A systematic review and meta-analysis.European Journal of Cancer 48: 3499–3513. doi: 10.1016/j.ejca.2012.05.001 22658807

[16] Chen L, Liu M, Bao J, Xia Y, Zhang J, Zhang L, et al. (2013) The correlation between apparent diffusion coefficient and tumor cellularity in patients: a meta-analysis. PLoS One 11(11):e79008. doi: 10.1371/journal.pone.0079008 24244402 PMC3823989

[17] Wilson DB, Lipsey MW (2001) The role of method in treatment effectiveness research: evidence from meta-analysis. Psychological methods 6: 413. 11778681

[18] Fan D, Liu S, Yang T, Wu S, Wang S, Li G, et al., (2014) Association between KIR polymorphisms and ankylosing spondylitis in populations: a meta-analysis. Mod Rheumatol 24(6):985–91. doi: 10.3109/14397595.2014.894489 24673577

[19] Fan D, Liu L, Ding N, Liu S, Hu Y, Cai G, et al. (2015) Male sexual dysfunction and ankylosing spondylitis: a systematic review and metaanalysis. J Rheumatol 42:252–7. doi: 10.3899/jrheum.140416 25448789

[20] Emmanuel Schneck, Edinger F, Hecker M, Sommer N, Pak O, Weissmann N, et al. (2020) Blood Levels of Free-Circulating Mitochondrial DNA in Septic Shock and Postsurgical Systemic Inflammation and Its Influence on Coagulation: A Secondary Analysis of a Prospective Observational Study. J Clin Med 9:2056. doi: 10.3390/jcm9072056 32629885 PMC7408641

[21] Jingxiu Fan, Jia Hu, Yingqiang Guo, Yan Kang (2020) Association of plasma mtDNA level with cardiopulmonary bypass-related inflammation in ICU patients. Acta Academiae Medicinae Militaris Tertiae 42:2403–2407.

[22] Enwei Guo, Dali Ren, Bingyu Zhang, Yang feng Yao Yulan, Ling Jia, et al. (2020) Change in amount of serum cell-free mitochondrial DNA and clinical relevance in trauma patients. J Surg Concepts Pract 25:315–321.

[23] Yi Jia, Xiaojie Dai, Sujuan Dong (2016) Correlation of mitochondrial DNA and inflammatory mediators in acute myocardial infarction patients. Journal of Cardiovascular & Pulmonary Diseases 35:606–608.

[24] Lam NY, Rainer TH, Chiu RW, Joynt GM, Lo YM (2004) Plasma mitochondrial DNA concentrations after trauma. Clin Chem 50(1):213–6. doi: 10.1373/clinchem.2003.025783 14709653

[25] Qin C, Liu R, Gu J, Li Y, Qian H, Shi Y, et al. (2015) Variation of perioperative plasma mitochondrial DNA correlate with peak inflammatory cytokines caused by cardiac surgery with cardiopulmonary bypass. J Cardiothorac Surg 10:85. doi: 10.1186/s13019-015-0298-6 26104758 PMC4479323

[26] Qin C, Gu J, Liu R, Xu F, Qian H, He Q, et al. (2017) Release of mitochondrial DNA correlates with peak inflammatory cytokines in patients with acute myocardial infarction. Anatol J Cardiol 17:224–228. doi: 10.14744/AnatolJCardiol.2016.7209 27721319 PMC5864983

[27] Qin C, Gu J, Qian H, Meng W (2016) Analysis of circulatory mitochondrial DNA level after cardiac surgery with cardiopulmonary bypass and potential prognostic implications. Indian Heart J 68:389–90. doi: 10.1016/j.ihj.2016.04.014 27316503 PMC4911438

[28] Yamanouchi S, Kudo D, Yamada M, Miyagawa N, Furukawa H, Kushimoto S (2013) Plasma mitochondrial DNA levels in patients with trauma and severe sepsis: time course and the association with clinical status. J Crit Care 28:1027–31. doi: 10.1016/j.jcrc.2013.05.006 23787023

[29] Wang HC, Lin YT, Hsu SY, Tsai NW, Lai YR, Su BY, et al. (2019) Serial plasma DNA levels as predictors of outcome in patients with acute traumatic cervical spinal cord injury. J Transl Med 17:329. doi: 10.1186/s12967-019-2084-z 31570098 PMC6771086

[30] Xishao Xie (2016) Associations of Mitochondria DNA content in Peritoneal Dialysis effulent with peritoneal solute tranport rate and peritonitis outcome in peritoneal dialysis[D]. Zhejiang province of China, Zhejiang university.

[31] Zhang JZ, Wang J, Qu WC, Wang XW, Liu Z, Ren JX, et al. (2017) Plasma mitochondrial DNA levels were independently associated with lung injury in elderly hip fracture patients. Injury 48:454–459. doi: 10.1016/j.injury.2017.01.009 28073488

[32] Bulgakova O, Kausbekova A, Kussainova A, Kalibekov N, Serikbaiuly D, Bersimbaev R (2021) Involvement of Circulating Cell-Free Mitochondrial DNA and Proinflammatory Cytokines in Pathogenesis of Chronic Obstructive Pulmonary Disease and Lung Cancer. Asian Pac J Cancer Prev;22(6):1927–1933. doi: 10.31557/APJCP.2021.22.6.1927 34181353 PMC8418868

[33] Cataño Cañizales YG, Uresti Rivera EE, García Jacobo RE, Portales Perez DP, Yadira B, Rodriguez Rivera JG, et al. (2018) Increased Levels of AIM2 and Circulating Mitochondrial DNA in Type 2 Diabetes. Iran J Immunol;15(2):142–155. 29947343 10.22034/iji.2018.39378

[34] Fan Z, Yang JY, Guo Y, Liu YX, Zhong XY (2022) Altered levels of circulating mitochondrial DNA in elderly people with sarcopenia: Association with mitochondrial impairment. Exp Gerontol;163:111802. doi: 10.1016/j.exger.2022.111802 35398474

[35] Gonçalves VF, Mendes-Silva AP, Koyama E, Vieira E, Kennedy JL, Diniz B (2021) Increased levels of circulating cell-free mtDNA in plasma of late life depression subjects. J Psychiatr Res;139:25–29. doi: 10.1016/j.jpsychires.2021.05.015 34022472

[36] Kageyama Y, Kasahara T, Kato M, Sakai S, Deguchi Y, Tani M, et al. (2018) The relationship between circulating mitochondrial DNA and inflammatory cytokines in patients with major depression. J Affect Disord;233:15–20. doi: 10.1016/j.jad.2017.06.001 28633757

[37] Lee JY, Lee DC, Im JA, Lee JW (2014) Mitochondrial DNA copy number in peripheral blood is independently associated with visceral fat accumulation in healthy young adults. Int J Endocrinol;2014:586017. doi: 10.1155/2014/586017 24707289 PMC3953665

[38] Peng Y, Zheng D, Zhang X, Pan S, Ji T, Zhang J, et al. (2019) Cell-Free Mitochondrial DNA in the CSF: A Potential Prognostic Biomarker of Anti-NMDAR Encephalitis. Front Immunol;10:103. doi: 10.3389/fimmu.2019.00103 30792710 PMC6375341

[39] Xu F, Liu RQ, Cao R, Guo LT, Zhang N, Huang K, et al. (2017) Perioperative plasma mitochondrial DNA dynamics and correlation with inflammation during infantile cardiopulmonary bypass. Indian Heart J;69(6):797–800. doi: 10.1016/j.ihj.2017.03.009 29174262 PMC5717290

[40] Zhong XY, Guo Y, Fan Z (2022) Increased level of free-circulating MtDNA in maintenance hemodialysis patients: Possible role in systemic inflammation. J Clin Lab Anal;36(7):e24558. doi: 10.1002/jcla.24558 35708020 PMC9279998

[41] Aswani A, Manson J, Itagaki K, Chiazza F, Collino M, Wupeng WL, et al. (2018) Scavenging Circulating Mitochondrial DNA as a Potential Therapeutic Option for Multiple Organ Dysfunction in Trauma Hemorrhage. Front Immunol 9:891. doi: 10.3389/fimmu.2018.00891 29867926 PMC5951958

[42] McIlroy DJ, Minahan K, Keely S, Lott N, Hansbro P, Smith DW, et al. (2018) Reduced deoxyribonuclease enzyme activity in response to high postinjury mitochondrial DNA concentration provides a therapeutic target for Systemic Inflammatory Response Syndrome. J Trauma Acute Care Surg 85:354–358. doi: 10.1097/TA.0000000000001919 30080781

[43] Gan L, Zhong J, Zhang R, Sun T, Li Q, Chen X, et al. (2015) The Immediate Intramedullary Nailing Surgery Increased the Mitochondrial DNA Release That Aggravated Systemic Inflammatory Response and Lung Injury Induced by Elderly Hip Fracture. Mediators Inflamm 2015:587378. doi: 10.1155/2015/587378 26273137 PMC4530272

[44] Zhang X, Wu J, Liu Q, Li X, Li S, Chen J, et al. (2020) mtDNA-STING pathway promotes necroptosis-dependent enterocyte injury in intestinal ischemia reperfusion. Cell Death Dis 11:1050. doi: 10.1038/s41419-020-03239-6 33311495 PMC7732985

[45] Timmermans K, Kox M, Scheffer GJ, Pickkers P (2016) Plasma Nuclear and Mitochondrial DNA Levels, and Markers of Inflammation, Shock, and Organ Damage in Patients with Septic Shock. Shock;45(6):607–12. doi: 10.1097/SHK.0000000000000549 26717107

[46] Deng X, Yang G, Zheng X, Yang Y, Qin H, Liu ZX, et al. (2020 ) Plasma mtDNA copy numbers are associated with GSTK1 expression and inflammation in type 2 diabetes. Diabet Med Nov;37(11):1874–1878. doi: 10.1111/dme.14132 31502701

[47] Zhang L, Deng S, Zhao S, Ai Y, Zhang L, Pan P, et al. (2016) Intra-Peritoneal Administration of Mitochondrial DNA Provokes Acute Lung Injury and Systemic Inflammation via Toll-Like Receptor 9. Int J Mol Sci 17:1425. doi: 10.3390/ijms17091425 27589725 PMC5037704

[48] Bao xianguo Sun Tiansheng (2015) The animal experimental research between mtDNA and lung damage relations on hip fracture[D]. Anhui province. Anhui medical university.

[49] Huang L, Chang W, Huang Y, Xu X, Yang Y, Qiu H (2020) Prognostic value of plasma mitochondrial DNA in acute respiratory distress syndrome (ARDS): a single-center observational study. J Thorac Dis 12:1320–1328. doi: 10.21037/jtd.2020.02.49 32395269 PMC7212167

[50] Miao Li (2015) The value of mitochondria DNA level to the severity and prognosis in children with sepsis[D]. Nanjing province. University of Nanjiang.

